# Severe neuromyelitis optica spectrum disorder induced by pucotenlimab: a case report and literature review

**DOI:** 10.3389/fimmu.2026.1783590

**Published:** 2026-04-30

**Authors:** Ziyu Zhang, Lankai Liao, Houfeng Zhou, Wen Xiang, Junyan Li

**Affiliations:** 1 Department of Pharmacy, Chengdu Fifth People’s Hospital, Chengdu, Sichuan, China; 2 Department of Emergency Medicine, Chengdu Fifth People’s Hospital, Chengdu, Sichuan, China; 3 Department of Thyroid and Breast Surgery, Chengdu Fifth People’s Hospital, Chengdu, Sichuan, China

**Keywords:** immune checkpoint inhibitors, immune-related adverse events, malignant tumor of breast, neuromyelitis optica spectrum disorder, pucotenlimab

## Abstract

Immune checkpoint inhibitors (ICIs) have been widely utilized in patients with cancer and established as a standard therapeutic modality for various solid tumors. Although generally well tolerated, ICIs can induce unique immune-related adverse events (irAEs) affecting systemic systems and organs, posing a significant challenge in clinical oncology practice. Neurological immune-related adverse events (N-irAEs) are relatively rare, but the clinical management of high-grade N-irAEs is extremely challenging and potentially life-threatening. Concurrently, the nonspecific manifestations and extensive differential diagnoses of N-irAEs render their diagnosis highly difficult, which may result in suboptimal clinical management. Herein, we report on a case of a patient with advanced breast cancer who developed severe adverse reactions subsequent to treatment with pucotenlimab and was diagnosed with anti-aquaporin 4 antibody (AQP4-Ab)-positive neuromyelitis optica spectrum disorder (NMOSD) following multidisciplinary discussion. Despite prompt administration of high-dose methylprednisolone and plasma exchange therapy, the patient’s neurological symptoms progressively worsened, leading to multiple organ dysfunction syndrome and subsequent death. A literature review on N-irAEs is also presented.

## 1 Introduction

Immunotherapy, by blocking immunosuppressive effects and inhibiting the functions of cytotoxic T-lymphocyte-associated antigen 4 (CTLA-4), programmed cell death protein 1 (PD-1), and its ligand programmed cell death-ligand 1 (PD-L1) (1), represents a major breakthrough in oncology over the past decade and has become a standard clinical treatment for various solid tumors. Immune-related adverse events (irAEs) are triggered by treatment with immune checkpoint inhibitors (ICIs). These events arise from the nonspecific activation of the immune system or the loss of autoimmune tolerance, which can affect nearly all organ systems and exhibit heterogeneity in their clinical manifestations, onset time, and severity (2). irAEs most commonly occur in the endocrine system, the skin, the lungs, and the gastrointestinal tract (3).

Neurological irAEs (N-irAEs) are relatively rare, with an estimated incidence of 1%–12%; however, their mortality rate is higher than that of other irAEs (4). Consequently, the National Comprehensive Cancer Network (NCCN) guidelines recommend that N-irAEs be classified as higher-grade events. The recognition and management of high-grade N-irAEs remain challenging, considering the risks of mortality and long-term disability. To date, there is a lack of strong scientific data to support the management of high-grade clinical forms (5). In addition, high-grade N-irAEs [Common Terminology Criteria for Adverse Events (CTCAEs) >II] are typically refractory to first-line corticosteroid therapy, posing great challenges to their clinical management; hence, timely initiation of second-line therapy is imperative (5). Therefore, timely identification and intervention of high-grade N-irAEs are crucial to potentially alter the prognosis of patients.

Pucotenlimab is a humanized immunoglobulin G4 (IgG4) monoclonal antibody that binds to programmed cell death protein 1 (PD-1) with high affinity and specificity and blocks its interaction with the ligands programmed cell death-ligand 1 (PD-L1) and PD-L2, thereby abrogating the PD-1 pathway-mediated immune suppression and restoring the antitumor immune activity of T cells (6). Pucotenlimab can be used as salvage therapy for metastatic triple-negative breast cancer (TNBC) and exhibits favorable efficacy and safety profiles (7). This article reports on a case of advanced invasive ductal breast cancer treated with eribulin plus pucotenlimab as salvage therapy, which was diagnosed as ICI-induced aquaporin 4 antibody (AQP4-Ab)-positive neuromyelitis optica spectrum disorder (NMOSD) after multidisciplinary team (MDT) consultation. Despite the prompt administration of high-dose methylprednisolone and plasma exchange therapy, the patient’s neurological symptoms progressively worsened, leading to multiple organ dysfunction and death. To better understand and manage ICI-induced N-irAEs, this article reviews the research advances in the epidemiology, potential pathophysiological mechanisms, diagnosis, and differential diagnosis of this disease.

## 2 Materials and methods

In this study, hematoxylin–eosin (HE) staining of pathological specimens of the patient’s breast mass and axillary lymph node mass was performed using a DAKO CoverStainer (Agilent Technologies, Glostrup, Denmark). Immunohistochemistry was conducted using DAKO PT Link48 and Leica BOND-III stainers (Leica Biosystems, Richmond, IL, USA). The reports were interpreted and reviewed by two pathologists from Chengdu Fifth People’s Hospital. Serum and cerebrospinal fluid (CSF) antibody detection reports were generated by the Chengdu Heimer Yunyin Center for Clinical Laboratory using cell-based assay (CBA) transfection and live cell-based assay (LCBA) methods, with interpretation by the center. Spinal cord magnetic resonance imaging (MRI) was performed using a Philips Achieva 1.5-T scanner (Philips Healthcare, Amsterdam, Netherlands) with contrast enhancement, and the reports were interpreted and reviewed by two radiologists from Chengdu Fifth People’s Hospital.

## 3 Case presentation

**Figure 1:**
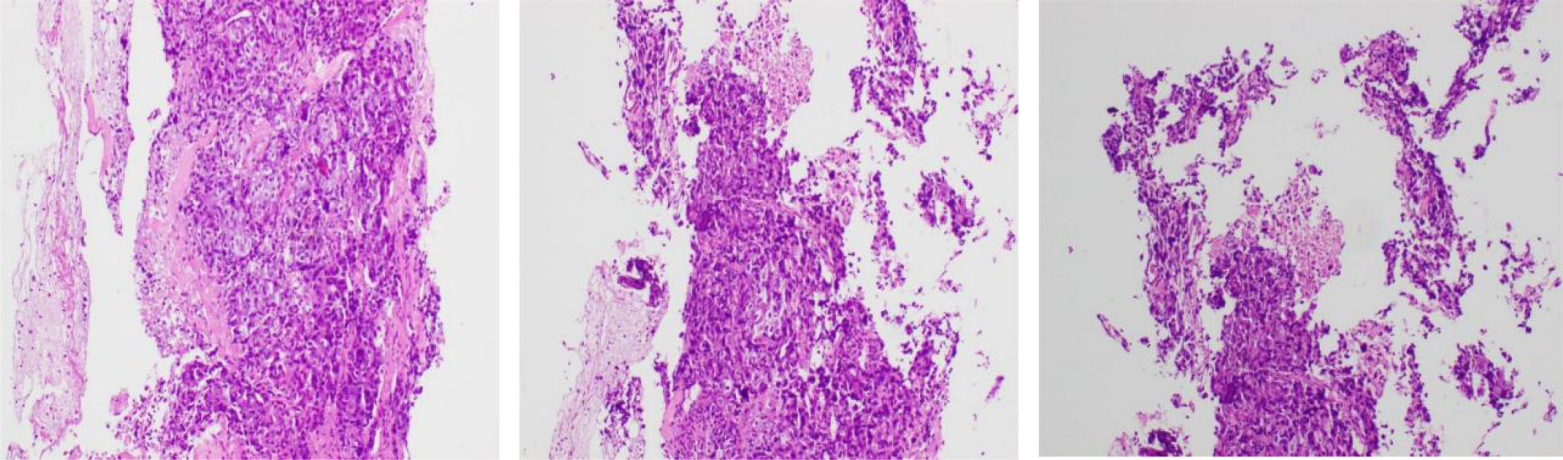
Pathological examination results with first diagnosed invasive ductal carcinoma.

A 56-year-old female patient presented to the outpatient department of Chengdu Fifth People’s Hospital in July 2023 with a left breast mass. Fine-needle aspiration biopsy (FNAB) of the left breast mass and axillary lymph nodes combined with immunohistochemistry confirmed the diagnosis of invasive ductal carcinoma of the left breast (cT3N2M0, stage IIIA, triple-negative) (the pathological diagnosis and immunohistochemistry are shown in **Figure 1**). She completed four cycles of neoadjuvant chemotherapy from July to September 2023, followed by left breast-conserving surgery plus axillary lymph node dissection in October 2023. Postoperative adjuvant chemotherapy and radiotherapy were subsequently administered from November 2023 to November 2024. A follow-up ultrasound in November 2024 revealed breast nodules and multiple cervical lymphadenopathies, for which excisional biopsy of the left cervical lymph nodes and resection of the left breast nodules were performed. Pathological examination confirmed lymph node metastasis of the primary breast lesion and malignant components in the breast nodules. Tumor metastasis was confirmed as left breast invasive ductal carcinoma (cTxN3M1, stage IV, triple-negative type). Salvage therapy with eribulin plus pucotenlimab was initiated on November 20, 2024.

**Figure 2:**
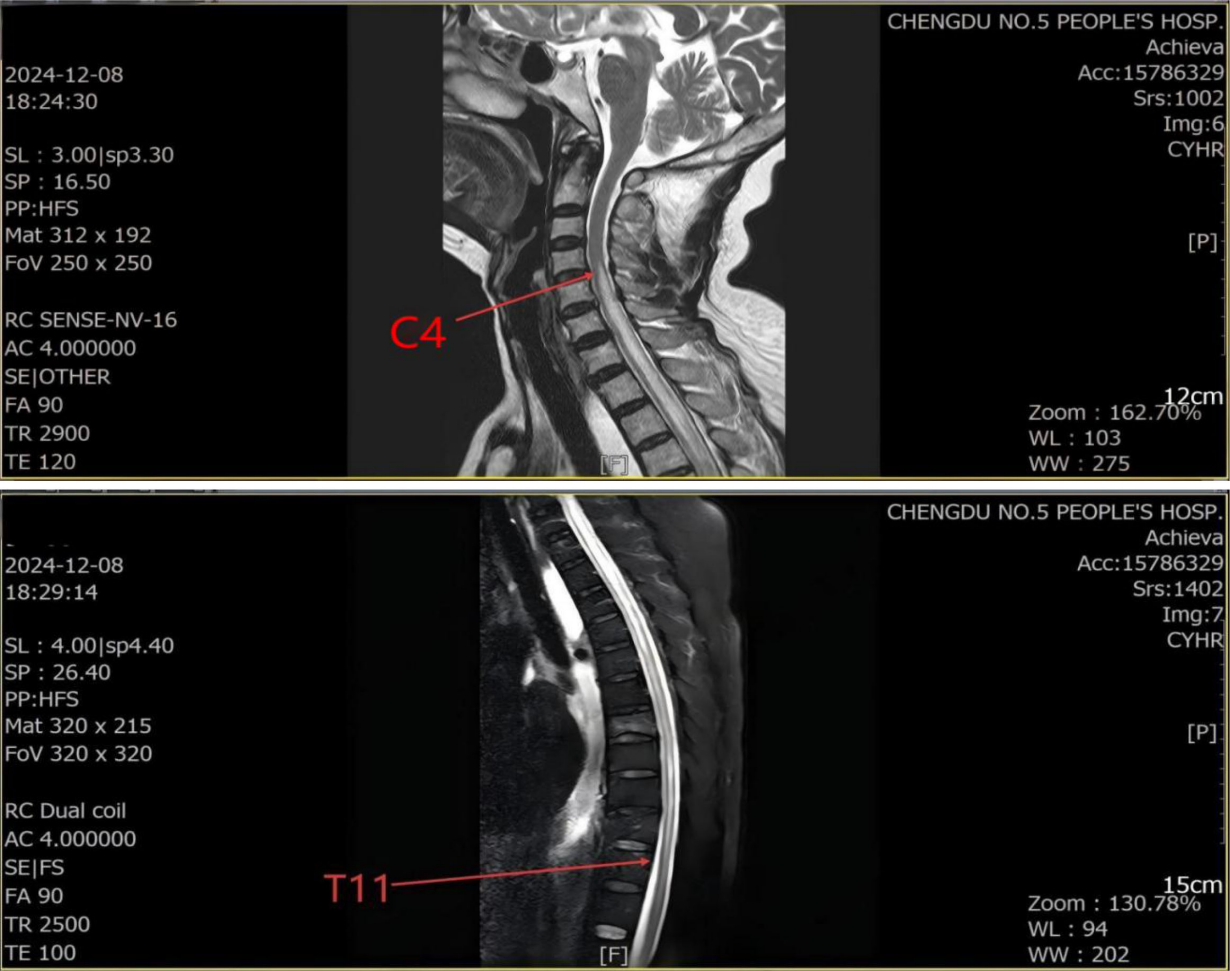
MRI report suggests: C4–T11 vertebral body plane spinal cord abnormal signal, the nature to be determined, considering the possibility of inflammatory lesions.

On December 3, 2024, the patient developed numbness and weakness of the lower extremities, abdominal pain, abdominal distension, and vomiting and was admitted to the hospital on December 6. Symptomatic and supportive treatments were administered, including potassium chloride injection to correct hypokalemia, mecobalamin for neurotrophic support, glycerin enema to relieve constipation, and probiotics to modulate intestinal flora. Despite initial symptomatic and supportive management, the neurological symptoms progressed. A neurology consultation on December 8 showed the results of neurological physical examination as follows: normal muscle strength of both upper extremities with symmetrically elicited tendon reflexes, loss of superficial sensation below the T3 spinal level, grade 0 muscle strength of both lower extremities, absent tendon reflexes of both lower extremities, and positive bilateral Babinski signs. Spinal MRI demonstrated longitudinally extensive transverse myelitis (LETM) (**Figure 2**). Testing for paraneoplastic antibody panel, demyelinating antibody panel, electromyography (EMG), and evoked potentials was recommended.

**Figure 3:**
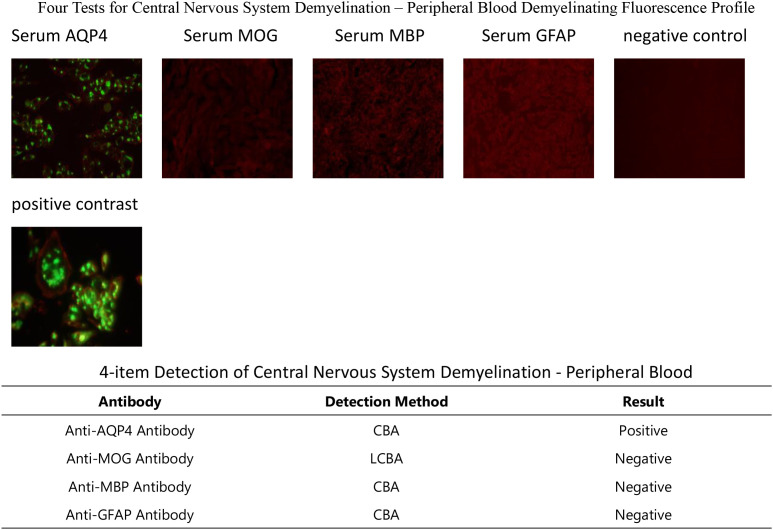
Positive anti-aquaporin 4 (AQP4) antibody in peripheral blood.

**Figure 4:**
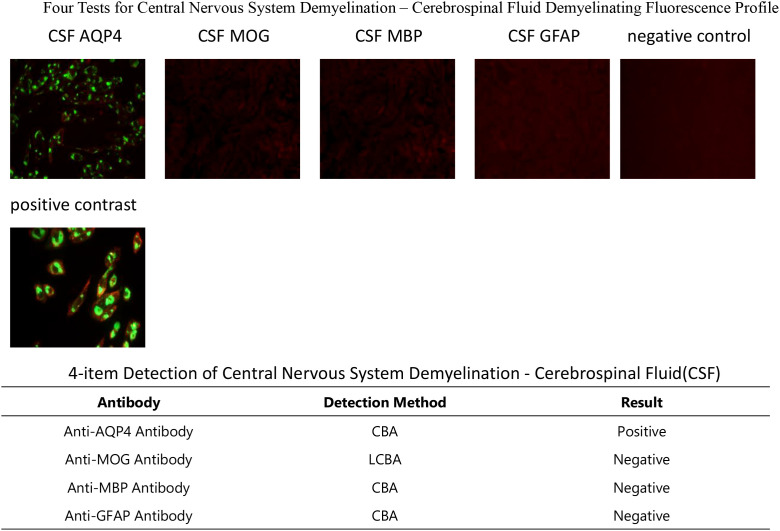
Positive anti-aquaporin 4 (AQP4) antibody in the cerebrospinal fluid (CSF).

**Figure 5:**
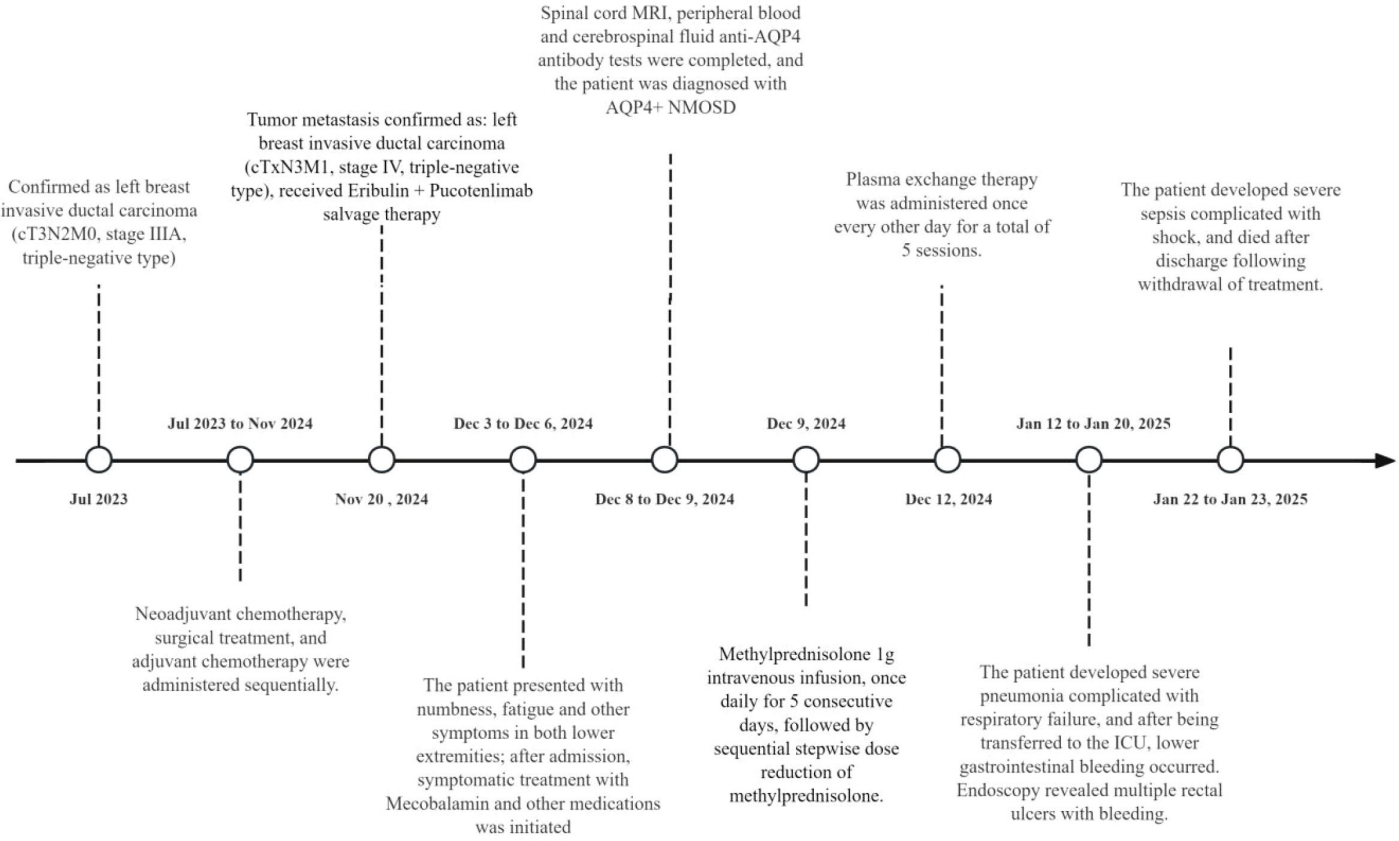
Patient course and treatment timeline.

On December 9, the serum and CSF tests showed negative paraneoplastic antibodies (**Table 1**), but positive anti-aquaporin 4 immunoglobulin G (anti-AQP4-IgG) (**Figures 3**, **4**). MDT involving the departments of breast surgery, oncology, neurology, nephrology, critical care, and clinical pharmacy confirmed the diagnosis of pucotenlimab-induced AQP4-positive NMOSD. High-dose methylprednisolone (1 g intravenously daily for 5 days, followed by gradual tapering) was initiated on December 9, and plasma exchange treatment (five courses, once every other day) was administered on December 12. Despite aggressive treatment, the neurological deficit symptoms persisted. On January 12, 2025, the patient developed severe pneumonia complicated with respiratory failure, which required endotracheal intubation, and was transferred to the intensive care unit (ICU). On January 20, lower gastrointestinal bleeding occurred, and endoscopy revealed multiple rectal ulcers with bleeding. On January 22, the patient developed septic shock requiring norepinephrine for blood pressure support. On January 23, the patient’s family elected to receive palliative care, and the patient died shortly after discharge. The timeline of the patient’s clinical course and treatment is shown in **Figure 5**.

**Table 1:** Results of the paraneoplastic syndrome antibody panel (peripheral blood).

Antibody tested	Detection method	Result
Anti-Hu antibody IgG	Western blotting	Negative
Anti-Yo antibody IgG	Western blotting	Negative
Anti-Ri antibody IgG	Western blotting	Negative
Anti-CV2 antibody IgG	Western blotting	Negative
Anti-amphiphysin antibody IgG	Western blotting	Negative
Anti-Ma1 antibody IgG	Western blotting	Negative
Anti-PNMA2 (Ma2/Ta) antibody IgG	Western blotting	Negative
Anti-SOX1 antibody IgG	Western blotting	Negative
Anti-Tr (DNER) antibody IgG	Western blotting	Negative
Anti-Zic4 antibody IgG	Western blotting	Negative
Anti-GAD65 antibody IgG	Western blotting	Negative
Anti-PKCγ antibody IgG	Western blotting	Negative
Anti-recoverin antibody IgG	Western blotting	Negative
Anti-titin antibody IgG	Western blotting	Negative
Anti-KLHL11 antibody IgG	Transfected cell assay	Negative

## 4 Discussion

### 4.1 Epidemiological overview of irAEs

Previous studies have shown that irAEs can involve various organs and tissues throughout the body (8, 9). The incidence of all-grade irAEs ranges from 65% to 76%, with grade 3 or higher irAEs occurring in 3%–5% of cases. Although the majority of adverse events are mild and reversible, severe fatal toxicities still account for 0.3%–1.3% of cases (10–12), representing one of the significant causes of unexpected death in patients with cancer. N-irAEs are relatively rare, but are potentially fatal complications associated with ICI therapy (13). A retrospective analysis of 59 clinical trials demonstrated that the incidence rates of N-irAEs were 3.8% for CTLA-4 inhibitors, 6% for PD-1 inhibitors, and 12.0% for combination therapy of the two immune checkpoint agents (14). The incidence of severe (grade 3–4) N-irAEs was 1.9% in patients treated with anti-CTLA-4 agents, 0.2%–0.4% in those treated with anti-PD-1 agents, and 0.1%–1% in those treated with anti-PD-L1 agents (15). N-irAEs predominantly occur during the induction phase of ICI therapy, with a median onset time of approximately 6 weeks (range, 1–74 weeks) after treatment initiation, of which 75%–80% develop within the first 4 months of treatment (16). Melanoma, younger age at treatment initiation, prior chemotherapy, and a history of surgery are also associated with an increased risk of N-irAEs (17). One study showed that the mortality rate of severe N-irAEs may reach 15% (18). Among these, encephalitis and myasthenia gravis are the most common fatal N-irAEs (19). The mortality rate of ICI-related myositis is approximately 22%, and it can exceed 50% when complicated by myocarditis or myasthenia gravis, classifying it as a fatal irAE (20, 21).

### 4.2 Pathophysiological mechanisms of N-irAEs

The core mechanism underlying neurological adverse events induced by ICIs is the breakdown of immune tolerance, which triggers aberrant immune responses specifically targeting self-neuronal antigens. The specific mechanisms involve multiple levels, including those of T cells, B cells, autoantibodies, and cytokines, and may be mediated through pathways such as molecular mimicry and the activation of preexisting subclinical autoimmune status. The main mechanisms can be summarized as follows:

*T-cell-mediated autoimmune response*: ICIs (e.g., anti-CTLA-4 and anti-PD-1/PD-L1 antibodies) abrogate the inhibition of T-cell activation by blocking the inhibitory signaling pathways on T cells (22, 23). These activated cytotoxic T cells can infiltrate the nervous system; attack neurons, neuroglial cells, or muscle tissues; and induce inflammation and damage (16).

*B-cell-mediated autoantibody production*: Activated T helper cells (in particular follicular helper T cells) can promote the proliferation and differentiation of B cells into plasma cells in the lymph nodes and induce the production of autoantibodies targeting neuronal antigens (24). These autoantibodies may attack the neuromuscular junction (e.g., acetylcholine receptors, leading to myasthenia gravis) and neuronal surface or intracellular antigens [e.g., Hu, Yo, and *N*-methyl-d-aspartate receptor (NMDAR)], thereby triggering antibody-mediated neuronal injury (24–26). In some cases, ICIs may potentiate preexisting subclinical autoantibody responses and drive them to pathogenic levels (22).

*Elevated levels of pro-inflammatory cytokines*: ICI therapy can induce a systemic inflammatory response, leading to increased levels of pro-inflammatory cytokines [e.g., interleukin-6 (IL-6), interferon-γ, and tumor necrosis factor-α, among others]. These cytokines may impair the integrity of the blood–brain barrier (BBB) or the blood–nerve barrier (BNB), facilitate the infiltration of immune cells and autoantibodies into the central and peripheral nervous system, and directly or indirectly induce neuroinflammation and neuronal damage (22).

*Molecular mimicry and cross-reactivity*: Tumor cells may express proteins homologous to the normal antigens of the nervous system (shared antigens). The immune response potentiated by ICIs, while attacking tumor cells, may misrecognize and attack nervous system tissues expressing homologous antigens, a mechanism known as molecular mimicry (27).

*Activation of preexisting subclinical autoimmunity*: Some patients may have preexisting autoimmune responses against neuronal antigens (e.g., low-titer autoantibodies or autoreactive lymphocytes) prior to ICI therapy, and these responses remain in a clinically quiescent state. ICI therapy abrogates immune suppression and may ignite or exacerbate such latent autoimmunity, leading to the onset of clinical symptoms (22).

AQP4-IgG-positive NMOSD may be a rare but severe N-irAE associated with ICI therapy. Its pathogenesis is directly linked to the abrogation of immune suppression and the disruption of autoimmune tolerance by ICIs, which may induce or unmask autoimmune responses targeting AQP4 (28). ICIs (e.g., anti-PD-1/PD-L1 and anti-CTLA-4 antibodies) enhance the antitumor immune response by blocking the inhibitory signaling pathways in T cells. This immunomodulatory process may also abrogate the suppression of autoreactive T and B cells, leading to the activation of autoreactive lymphocytes (including AQP4-targeted B and T cells), thereby inducing the production of pathogenic AQP4-IgG. The antibodies then attack astrocytes in the central nervous system (CNS), triggering the clinical manifestations of NMOSD (28–30). In addition, studies have indicated that ICIs may trigger paraneoplastic syndromes associated with anti-Hu antibodies and other autoantibodies, suggesting that they may also induce AQP4-targeted immune responses through these similar mechanisms (31).

### 4.3 Diagnosis, differential diagnosis, and related case review of NMOSD

NMOSD is a group of autoimmune-mediated inflammatory demyelinating diseases of the CNS predominantly affecting the optic nerves and the spinal cord (32). The pathogenesis of NMOSD is primarily associated with AQP4 antibodies, representing an independent disease entity clinically and pathogenetically distinct from multiple sclerosis (MS) (33). Approximately 70%–80% of patients with NMOSD are serologically AQP4-IgG-positive (34). Thus, AQP4-IgG serves as a highly specific diagnostic marker, with a specificity of up to 90% and a sensitivity of approximately 70% (35). Clinically, diagnosis is based on the fundamental criteria of “medical history + core clinical manifestations + imaging features + biomarkers” and is stratified by the AQP4-IgG status, further supplemented by other subclinical and immunological evidence. For the specific diagnostic criteria of NMOSD, please refer to the criteria formulated by the International Panel for NMOSD Diagnosis (IPND, 2015) (32). The clinical data of this patient fully met the aforementioned diagnostic criteria, confirming the definitive diagnosis of NMOSD. The patient’s Naranjo score was 5, indicating that the occurrence of NMOSD in this patient was probably related to the administration of ICIs. Paraneoplastic syndromes may present with similar clinical manifestations and imaging features; however, the results of testing of the 15 paraneoplastic syndrome-related autoantibodies in the patient’s peripheral blood were negative, making the diagnosis of paraneoplastic syndromes less likely. Certainly, complete differentiation between these two entities is extremely challenging. Reviews have shown that ICIs themselves can induce the onset and progression of paraneoplastic syndromes as their pathophysiological mechanisms and etiology lead to the same outcome despite different pathways. Studies have reported that ICIs can induce neurological adverse events similar to paraneoplastic neurological syndromes. In addition, some articles have suggested that AQP4 is expressed in the neural tissue regions of ovarian teratomas and that AQP4 antibodies may also serve as novel associated antibodies for paraneoplastic syndromes (22, 36). In this patient, testing for AQP4 antigen expression in the tumor tissue was not performed prior to ICI administration. Therefore, rigorous differentiation between these two entities is unnecessary in clinical practice, given the fact that their main therapeutic approaches are essentially identical, and emphasis should be placed on early identification, timely intervention, and treatment. In addition, the possibility of a preexisting subclinical NMOSD or the coincidental occurrence of the disease in the patient cannot be excluded. Nevertheless, based on the patient’s response to steroid therapy and poor prognosis, combined with research evidence suggesting that patients with high-grade N-irAEs induced by ICIs tend to have a poorer response to steroid therapy and an unfavorable prognosis (5, 22), we favor the final diagnosis of ICI-induced high-grade N-irAEs in this patient. Concurrently, some studies have recommended screening for paraneoplastic neurological syndrome antibodies prior to the initiation of ICI therapy, which is worthy of exploration and implementation (22). This approach can better prevent high-risk patients from receiving ICI treatment. To date, a small number of studies have reported the treatment and prognosis of NMOSD cases associated with other ICIs (see **Table 2**).

**Table 2:** Clinical characteristics of immune checkpoint inhibitor (ICI)-associated neuromyelitis optica spectrum disorder (NMOSD) cases.

Age/sex	Tumor type	ICI administered	Treatment regimen	AQP4 antibody status	Outcome	Reference
75/male	Squamous cell lung carcinoma	Nivolumab	Corticosteroids, plasma exchange	AQP4-Ab (+) (serum)	Not improved	Narumi et al. (37)
63/female	Lung adenocarcinoma	Pembrolizumab	Intravenous methylprednisolone 1,000 mg/day for 3 days, followed by oral prednisolone 0.5 mg kg^−1^ day^−1^ and plasma exchange	AQP4-Ab (+) (serum)	Improved	Shimada et al. (38)
57/male	Uveal melanoma of the right eye	Nivolumab plus ipilimumab	Intravenous methylprednisolone 1,000 mg for 5 days, followed by oral prednisone tapering over 10 weeks with symptom improvement	AQP4-Ab (+) (serum)	Improved	Khimani et al. (39)
81/female	Clear cell renal cell carcinoma	Nivolumab	High-dose intravenous methylprednisolone combined with 5 cycles of plasma exchange	AQP4-Ab (+)	Death	Weiss et al. (40)
54/female	Multiple basal cell carcinomas	Cemiplimab	High-dose methylprednisolone (1 g/day) for 5 days, followed by tapering; subsequent plasma exchange administered due to no significant improvement	AQP4-Ab (+)	Improved	Oelbrandt et al. (41)
47/male	Advanced lung adenocarcinoma	Atezolizumab	A regimen of five doses of 1 g methylprednisolone, accompanied by five sessions of alternate-day plasmapheresis. Following hospital discharge, continued immunosuppression with weekly administration of tocilizumab (162 mg)	AQP4-Ab (+)	Improved	Pedrero Prieto et al. (42)

## 5 Conclusion

This article reports on a case of AQP4-positive NMOSD induced by pucotenlimab in a patient with invasive ductal breast cancer and reviews the relevant literature on N-irAEs. With the widespread clinical application of ICIs, although ICI-associated AQP4-positive NMOSD is relatively rare, it has become an unavoidable clinical challenge that must be addressed. Healthcare providers must strengthen the monitoring and management of ICI-associated N-irAEs during ICI therapy to ensure the safety and quality of patient treatment.

## Data Availability

The original contributions presented in the study are included in the article/Supplementary Material. Further inquiries can be directed to the corresponding author.
